# Anesthesiologists as perioperative hospitalists and outcomes in patients undergoing major urologic surgery: a historical prospective, comparative effectiveness study

**DOI:** 10.1186/s13741-018-0090-y

**Published:** 2018-06-19

**Authors:** Gary Stier, Davinder Ramsingh, Ronak Raval, Gary Shih, Bryan Halverson, Briahnna Austin, Joseph Soo, Herbert Ruckle, Robert Martin

**Affiliations:** 1 Anesthesiology, Internal Medicine and Critical Care, Loma Linda University Medical Center, 11234 Anderson Street, MC-2532-D, Loma Linda, CA 92354 USA; 2 Department of Anesthesiology, Loma Linda University Medical Center, 11234 Anderson Street, MC-2532-D, Loma Linda, CA 92354 USA; 3 Department of Urology, 11234 Anderson Street, MC-2532-D, Loma Linda, CA 92354 USA

## Abstract

**Background:**

Perioperative care has been identified as an area of wide variability in quality, with conflicting models, and involving multiple specialties. In 2014, the Loma Linda University Departments of Anesthesiology and Urology implemented a perioperative hospitalist service (PHS), consisting of anesthesiology-trained physicians, to co-manage patients for the entirety of their perioperative period. We hypothesized that implementation of this PHS model would result in an improvement in patient recovery.

**Methods:**

As a quality improvement (QI) initiative, the PHS service was formed of selected anesthesiologists who received training on the core competencies for hospitalist medicine. The service was implemented following a co-management agreement to medically manage patients undergoing major urologic procedures (prostatectomy, cystectomy, and nephrectomy). Impact was assessed by comparisons to data from the year prior to PHS service implementation. Data was compared with and without propensity matching. Primary outcome marker was a reduction in length of stay. Secondary outcome markers included complication rate, return of bowel function, number of consultations, reduction in total direct patient costs, and bed days saved.

**Results:**

Significant reductions in length of stay (*p* <  0.05) were demonstrated for all surgical procedures with propensity matching and were demonstrated for cystectomy and nephrectomy cases without. Significant reductions in complication rates and ileus were also observed for all surgical procedures post-PHS implementation. Additionally, reductions in total direct patient costs and frequency of consultations were also observed.

**Conclusions:**

Anesthesiologists can safely function as perioperative hospitalists, providing appropriate medical management, and significantly improving both patient recovery and throughput.

**Electronic supplementary material:**

The online version of this article (10.1186/s13741-018-0090-y) contains supplementary material, which is available to authorized users.

## Summary key points

### Question

After receiving core competency training for hospitalist medicine, are perioperative providers able to perform as hospitalists for patients undergoing urologic surgery?

### Findings

Reductions in complication rates and length of stay were observed after implementation of an anesthesiology supported perioperative hospitalist service.

### Meaning

Anesthesiologists can safely function as perioperative hospitalists for patients undergoing urologic surgery.

## Background

In the United States (U.S.), health care has been a subject of great debate and it has been stated that we are facing a crisis in both the quality and the cost of delivered care (Kain et al. 2014). Perioperative care, in particular, has been identified as an area of high cost with a wide variability in quality (Kain et al. 2014; Lilot et al. 2015). There are a multitude of factors that have been suggested to account for this, including lack of care coordination, unwarranted lengths of stay, excess readmissions, and wide dissimilarity in perioperative patient management (Lilot et al. 2015; Barry et al. 2011; Desebbe et al. 2016; Ghaferi et al. 2009, 2011; Ravikumar et al. 2010). Current management strategies in the perioperative setting, along with the responsibility of *medical management* of patients throughout the perioperative period, are highly variable across various health care practice environments.

Recently, anesthesiologists in the U.S. have investigated a larger role in the patient’s surgical experience as part of the concept of the perioperative surgical home (PSH), which is a patient-centered, physician led, interdisciplinary, and team-based system of coordinated care (Kain et al. 2014). Similarly, the concept of Enhanced Recovery After Surgery (ERAS), which is the development of evidence-based care pathways for the enhancement of perioperative care, has demonstrated significant improvement in patient care (Ljungqvist et al. 2017). While the concepts of PSH and ERAS provide the tools to improve perioperative care, the decision of which, and how, various health care providers should be involved remains unresolved. In addition, the question of which provider should be medically managing these patients is also of debate.

In the early 1980s, the concept of a group of physicians who exclusively practice inpatient medicine began, but the term “hospitalists” was not coined until 1996 (Wachter and Goldman 1996). Today, the concept has formalized into an accepted physician role and national societies have formed. One such society is the Society of Hospitalist Medicine (SHM), which has suggested that perioperative patient care is one of the “foundations of hospital medicine” and has published practice guidelines for perioperative care (Medicine. SoH 2008). Over time, the level of collaboration between hospitalists and surgeons has continued to grow, with more hospitalists now referring to themselves as perioperative physicians (Macpherson et al. 1994; Adesanya and Joshi 2007; Merli 2004). In the U.S., it is a common practice for the hospitalist to play a vital role in the surgical experience, filling a void between office-based internists, surgeons, and anesthesiologists (Adesanya and Joshi 2007). Indeed, the implementation of co-management strategies between hospitalists and both orthopedic and cardiothoracic surgeons has demonstrated success (Macpherson et al. 1994; Merli 2004; Huddleston et al. 2004).

As models of coordinated care continue to be implemented, the role of the hospitalists for perioperative care will continue and may even grow. Although hospitalists traditionally have had specialty training in Internal Medicine, Family Medicine, or Pediatrics, the role of hospitalists in perioperative care is not exclusive to these specialties. Moreover, the American Board of Physician Specialties does not exclude anesthesiologists applying for certification in hospitalist medicine. Anesthesiologists in the U.S. are well suited as perioperative hospitalists, as the majority of core competencies listed by the SHM are covered during internship (PGY-1) and anesthesiology residency training (PGY2-4).

Based on this concept, in the early part of 2014, the Loma Linda University (LLU) Department of Anesthesiology sought to implement a *perioperative hospitalist service* (PHS), consisting of anesthesiology-trained physicians, to co-manage patients undergoing urologic surgery for the entirety of their perioperative period. The purpose of this service was to coordinate care and medically manage patients throughout the entire perioperative period, i.e., from the decision to operate until discharge from the hospital. We hypothesized that an effective implementation of this PHS model would result in an improvement in patients throughout, hospital resource utilization, complication rate, recovery, and length of stay (LOS) when compared to the year prior to service implementation.

## Methods

The study was approved by the Institutional Review Board at Loma Linda University (IRB #5160253). Patient consent was waived as this was a quality improvement initiative. Since the study was initiated as a quality improvement (QI) project, it is reported following the Standards for Quality Improvement Reporting Excellence (SQUIRE guidelines) (Davidoff et al. 2009; Ogrinc et al. 2008) and is presented as a historical prospective comparative effectiveness format following the GRACE (Good Research for Comparative Effectiveness) initiative principles and checklist (Dreyer 2013; Dreyer et al. 2010).

From September 2015 to July 2016, all patients undergoing the following urologic procedures at Loma Linda University Surgical Hospital were initially included in the QI project: partial or radical nephrectomy, cystectomy, and prostatectomy. This hospital is a 25-bed acute care surgical hospital with a 4-bed intensive care unit that is adjacent to the main hospital, which is a tertiary university medical center. Prior to the PHS service, the urology service was the primary for postoperative management. Baseline data was taken from retrospective data analysis of the same urologic procedures performed at this hospital in 2014. Patients less than 18 years of age, pregnant women, and emergency surgery were excluded from the study.

### Development of the quality improvement initiative

**Fig. 1:**
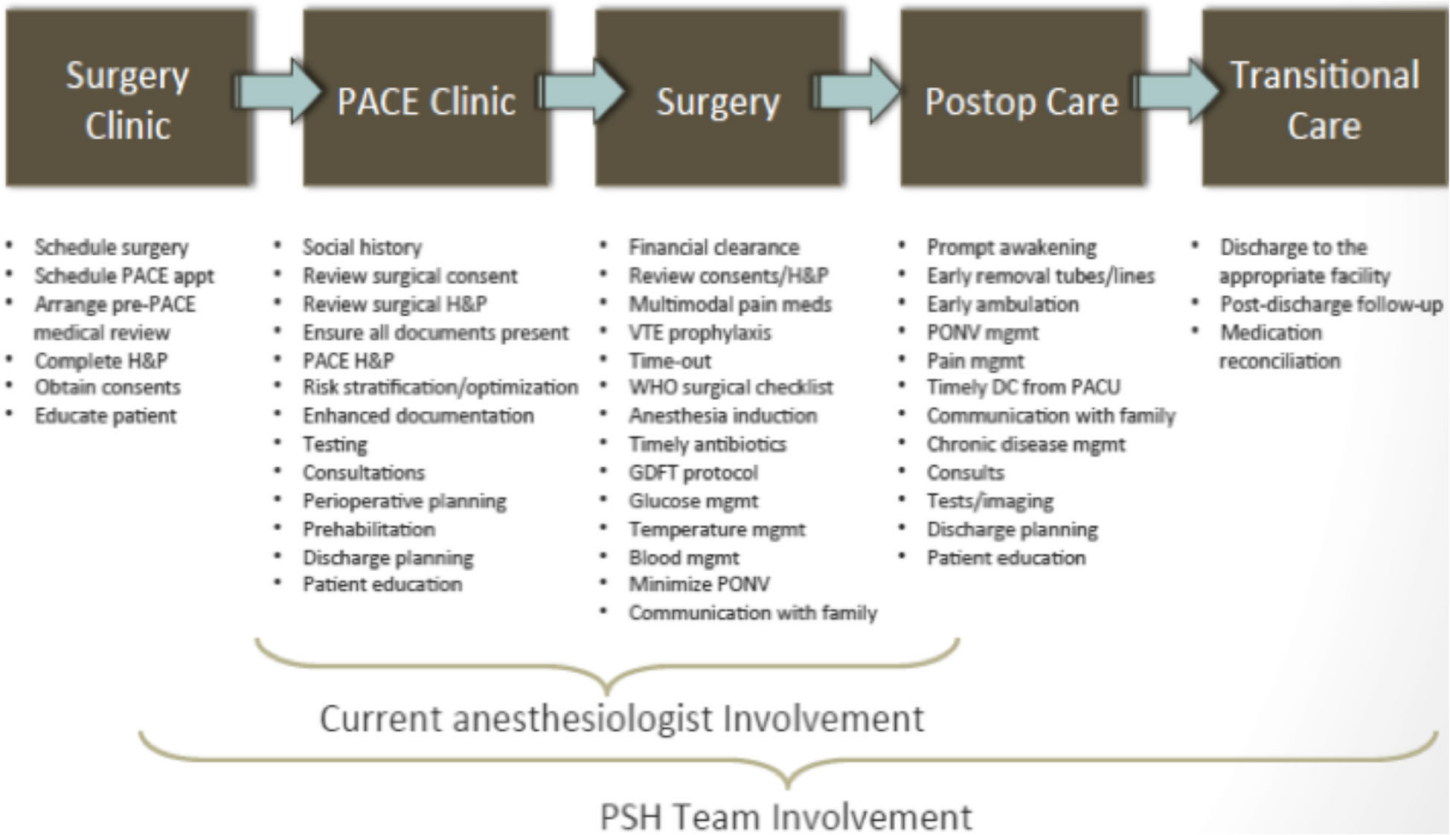
Roles of the perioperative hospitalist service. The diagram highlights the roles of anesthesiologists both prior to and post-implementation of the perioperative hospitalist service

Initial discussions occurred in mid-2014 between the Departments of Anesthesiology and Urology regarding the benefits of instituting a service of perioperative hospitalists whose focus would be to integrate all aspects of preoperative, intraoperative, and postoperative care. Once an agreement was reached on the merits of the partnership, a PHS planning team was assembled, consisting of three surgeons, three anesthesiologists, a hospital administrator, a nurse administrator, a nurse practitioner, a case manager, two patient safety officers, and an information technology expert. The goal of the PHS program was to improve perioperative care delivery and lower hospital cost for patients undergoing major urologic surgery. The focus of the PHS services was for the anesthesiology team members to serve as anesthesiology hospitalists providing daily medical care management as well as perioperative consultation services. Between September 2014 and December 2014, multiple meetings occurred to determine primary PHS team members, specific duties and activities of the PHS team, lines of authority, the creation of clinical pathways, and care plans intended to reduce practice variation, data capture and analytics, and clinical outcome targets. An outline of the roles of the PHS service during each of the phases of surgical care is shown in Fig. 1. Importantly, all anesthesiology department team members who rotate at this hospital (64 attending anesthesiologists, 45 residents, and 56 nurse anesthetists) were included in the intraoperative management of this study with consultative support of PHS service.

Implementation of the PHS program occurred over a 6-month period (January–June 2015). During this period, termed the *transition phase*, additional PHS faculty were recruited and trained. The period of July 2015 to September 2016 was the “study phase” of this QI project during which clinical impact was evaluated. The daily postoperative PHS multidisciplinary care team consisted of one anesthesiology attending, one anesthesiology resident physician, a department of urology nurse practitioner, hospital-specific case manager-discharge planner, bedside nurse, unit charge nurse, respiratory therapist, and a department of urology resident physician. Prior to PHS implementation, this team did not include an anesthesiology attending or resident.

### Designing the intervention: the perioperative hospitalist service

The anesthesiology PHS program faculty members consisted of five anesthesiologists, including three faculty with subspecialty training in critical care medicine. The core competencies for hospitalists published by SHM were used as a reference to ensure the appropriate level of skill and training. This publication outlines 19 clinical conditions as the core competencies for hospitalists (Table 1) (The core competencies in hospital medicine 2006; Dressler et al. 2006). Of these, 15 of the 19 clinical conditions are listed as part of the core competencies for anesthesiology training (ABA.org 2016). The four clinical conditions that do not overlap are cellulitis, gastrointestinal bleeding, stroke, and urinary tract infection. For the competencies not addressed during anesthesiology residency training, the group received additional education and training to obtain proficiency during the transition phase described above. Specifically, the director of PHS service, who is board certified in internal medicine and anesthesiology, developed educational material that targeted each of the four conditions. Review of these materials was required during the transition period and was discussed with the PHS director during regular meetings. In addition, the PHS director identified and discussed cases with members during the transition period that highlighted the management of each of these conditions. Finally, the PHS director reviewed selected patient records during the initial 3 months of the transition period to evaluate and educate on the appropriate management decisions related to the above clinical conditions.

**Table 1:** Perioperative Hospitalist: clinical conditions

Perioperative hospitalist: clinical conditions	
Acute coronary syndrome	
Acute kidney injury	
Alcohol and drug withdrawal	
Asthma	
Cardiac Arrhythmia	
Cellulitis	
Chronic obstructive pulmonary disease	
Community-acquired pneumonia	
Congestive heart failure	
Delirium and dementia	
Diabetes mellitus	
Gastrointestinal bleed	
Hospital-acquired pneumonia	
Pain management	
Perioperative medicine	
Sepsis syndrome	
Stroke	
Urinary tract infection	
Venous thromboembolism	

The table lists the 19 clinical conditions identified as the core competencies for hospitalists

Based on significant allocation of departmental resources (removal of one attending faculty, every day, from the operating room), the PHS service was developed in the following manner: One of the five anesthesiologists identified for the PHS service would take 1 week (7 days) of PHS coverage. This included weekday (Monday through Friday) rounds on all patients undergoing the three surgical procedures listed. The same anesthesiologists would have 24/7 pager call responsibilities for this week of PHS coverage. During the weekend (Saturday and Sunday), the PHS attending would be on pager call in a similar manner and saw patients in person, as per consultation by the in-house urology resident or urology attending. All holidays for the entirety of the QI period were covered in a similar manner as described for weekend coverage. Specific responsibilities for the PHS team are listed below.

### Preoperative phase

All patients initially seen in the urology clinic for the above surgical procedures were given an educational pamphlet, which described the perioperative care process and identified the PHS multidisciplinary team. After review by the PHS team, a consultation note was completed and recommendations were shared with the preoperative assessment clinic care team prior to the scheduled appointment date. All patients involved in this study were seen in the LLU preoperative assessment clinic and evaluated by either an anesthesiology resident or a nurse practitioner under the supervision of the PHS attending. Patients deemed to be at particularly high risk by the surgeons underwent a separate preoperative consultation by the PHS attending on the day of their urology visit. This consultation note included recommendations for preoperative, intraoperative, and postoperative management.

In addition, current evidence-based preoperative assessment tools (Table 2) were made available to be utilized during the preoperative assessment. These enhanced risk assessment tools (Table 2) were employed to better anticipate and manage perioperative medical issues that might adversely impact care quality and patient flow (e.g., nutritional risk, delirium risk, and frailty). Evaluation on the use of these tools was not captured for this project.

**Table 2:** Perioperative risk tools

Perioperative risk tools	
Obstructive sleep apnea: STOP-Bang Questionnaire	
Postoperative nausea and vomiting (PONV): Apfel score	
Cardiac ischemic risk: Revised Cardiac Risk Index (RCRI)	
Angina pectoris grade: Canadian Cardiovascular Angina Grade (I–IV)	
Heart failure risk: New York Heart Association Functional Classification (I–IV)	
Hopkins frailty score	
Postoperative delirium risk	
Cognitive impairment risk: Modified Mini-Cog Examination	
Nutritional risk	
Chronic pain syndrome	

The table lists enhanced risk assessment tools that were made available with the implementation of the perioperative hospitalist service

### Intraoperative phase

Intraoperative management was left to the discretion of the primary anesthesia team. The PHS team was always available for intraoperative consultation regarding patient care.

### Postoperative phase

Postoperative care was managed and coordinated by the anesthesiology PHS team and represented the majority of PHS implementation efforts. The PHS team conducted daily morning rounds Monday through Friday, with the PHS attending anesthesiologist available by pager 24/7 during the week as described above. All medical issues were directed and managed by the PHS attending, including pain management and medical management of all chronic diseases (Table 1). Specialty consultations were requested at the discretion of the PHS attending. The PHS anesthesiology hospitalist and surgery team jointly made decisions regarding blood transfusions, anticoagulation management, imaging studies, and nutritional therapy. Multimodal pain management strategies were formulated preoperatively and continued postoperatively. Emphasis was placed on early withdrawal of intravenous opioid medications, with focus directed to early institution of non-opioid pain medication. Non-steroidal anti-inflammatory drug therapy was emphasized for pain management, but were withheld for serum creatinine ≥ 1.5 mg/dL, and replaced with intravenous or oral opioids. Postoperative ambulation was targeted to occur by postoperative day one. Oral nutritional therapy was started as soon as there was evidence of return of bowel function (active bowel sounds, flatus). For patients undergoing radical cystectomy procedures, alvimopan was utilized to facilitate earlier return of bowel function.

#### Outcome measurements

To evaluate the impact of the PHS on patient care, we compared the pre-implementation period (1/2014 through 10/2014 dates) to the post-implementation period (9/2015 to 7/2016). After approval from the IRB, outcome data were collected retrospectively using our electronic medical record and billing records. In order to guarantee that data acquisition was the same in the two time periods, clinical data was recorded retrospectively in both time periods even though the PHS program was implemented prospectively as per GRACE and SQUIRE guidelines (Dreyer 2013; Dreyer et al. 2010).

#### Process measures

To ensure equality between the representative datasets, the ICD-09 procedural codes that were identified for patients seen by the PHS service was used to gather the list of patients who received similar procedures the year prior to PHS implementation. Retrospective data retrieval was gathered by the two separate teams (Anesthesiology and Urology), and datasets were compared for homogeneity. The urology team also reviewed operative records to evaluate for changes in surgical practice.

### Outcome measures

The primary outcome measure was LOS in the hospital after surgery, defined as the number of nights spent in the hospital after the day of surgery. Criteria for hospital discharge included stability of vital signs with no fever, control of postoperative pain, absence of other postoperative complications, and ability to function at home independently or with the home care provided.

Secondary outcome measures included change in average bed days per patient, percent change in total direct cost per discharge, incidence of postoperative complications, specialty consultations (frequency), return of bowel function (defined as postoperative day for return of flatus), and readmission to the hospital within 30 days. Change in average bed days were calculated as the average LOS in the PHS group minus LOS in the pre-PHS. Total direct cost per patient data was derived from the hospital’s final database for the surgical procedures listed. Total direct cost per patient was composed of total fixed and variable direct cost. Average percent change was calculated by comparing average cost from the post-PHS category to the pre-PHS category. Consultations were calculated by reviewing the number of consultation orders placed for each patient during the study period. Specific perioperative complications reviewed included acute kidney injury (defined as 1.5× increase in baseline serum creatinine), deep venous thrombosis, myocardial infarction, pneumonia, pulmonary embolism, sepsis, stroke, surgical site infection, urinary tract infection, erectile dysfunction, and ileus. Ileus was defined as any documented event of intolerance of diet, abdominal distension, nausea, or vomiting. All other complications were identified by examining problem list in progress notes and in discharge summary notes. Information was collected from review of electronic medical record (Epic Systems Corporation, Verona, WI).

### Analysis

To accurately present the impact of this QI project, we have conducted two sets of analysis on the data: one with propensity analysis and one with the unmatched raw data. Our propensity model matched urologic procedures before the implementation of PHS (2014) to post-PHS implementation (2015 and 2016). For all procedures in this study, the propensity scores generated were based on the following characteristics: age, weight, height, and ASA Scores. The covariates of interest were chosen based on historical knowledge of baseline differences in urologic procedures and expert opinion as previously supported (Austin 2007). 1:1 nearest neighbor matching was conducted with a caliper 0.15 as per previously suggested (Austin 2007). For this dataset, 1:1 ratio matching was chosen despite its reduction in samples available, in an effort to keep statistical power up, due to the potential bias of the treatment effect with 1:2 matching (Austin 2007).

Continuous variables were summarized using both median and percentile ranges (25/75). Propensity analysis was conducted on baseline characteristics followed by matching using the propensity scores. The degree to which matched propensity scores resulted in a matched sample was measured using standardized difference scores and balance distribution tables. The Wilcoxon paired test was used for comparisons between continuous variable after propensity analysis. For non-paired data, the Mann Whitney test was used for comparisons between continuous variables. The Fisher’s exact test was used to analyze count data. All analyses were performed with R (version 3.2.1).

## Results

In the pre-PHS period, 163 subjects (72 prostatectomy, 65 nephrectomy, 26 cystectomy) were reviewed. In the post-PHS period, 261 subjects (125 prostatectomy, 90 nephrectomy, 46 cystectomy) were reviewed (Tables 3A and 4A). Details regarding the number of subjects and comparison between groups after 1:1 propensity matching are shown in Tables 3B and 4B. A total of seven surgeons were involved in the care of the patients throughout this time period with no change in surgical volume. Review of the operative records by the urology team indicated no change in surgical practice over the project period (2014 to 2016). Propensity balance tables (Additional file 1) showed absolute standardized differences of less than 2% in all demographic categories between groups after matching (Table 3B). Operative time for prostatectomy cases was significantly less (*p* <  0.001) in the post-PHS group for both the propensity matched and unmatched datasets. Additionally, the propensity matched cystectomy patients showed a significant decrease in the total intraoperative IV fluids administered.

**Table 3:** Demographic data and comparisons

		Prostatectomy pre-program	Prostatectomy post-program	*p* values	Nephrectomy pre-program	Nephrectomy post-program	*p* values	Cystectomy pre-program	Cystectomy post-program	*p* values
A: Unmatched data comparisons										
*N*		71	125		65	90		26	46	
Age	Median (25/75 interval)	64 (59, 69)	65 (60, 71)	0.656	65 (56, 72)	60 (53, 70)	0.643	71 (62, 78)	71 (66, 79)	0.982
Height	Median (25/75 interval)	174 (171, 179)	176 (171, 182)	0.323	167 (158, 175)	169 (159, 176)	0.949	173 (167, 179)	167 (167, 177)	0.605
Weight	Median (25/75 interval)	88 (79, 100)	89 (81, 98)	0.694	86 (74, 98)	81 (70, 94)	0.67	80 (65, 95)	81 (69, 94)	0.994
ASA	Median (25/75 interval)	2 (2, 3)	2 (2, 3)	1	2 (2, 3)	2 (2, 3)	1	3 (3, 3)	3 (2, 3)	0.999
IV Fluid (ml)	Median (25/75 interval)	1887 (1500, 2300)	1700 (1400, 2000)	0.069	2500 (1850, 3032)	2175 (1738, 2808)	0.135	3450 (2658, 4050)	3250 (2038, 3488)	0.070
EBL (ml)	Median (25/75 interval)	100 (100, 200)	100 (100, 175)	0.829	150 (56, 200)	100 (50, 225)	0.755	250 (107, 437)	625 (300, 850)	0.364
Operative Time (Minutes)	Median (25/75 interval)	332 (294, 366)	302 (268, 338)	*< 0.001*	262 (226, 333)	285 (228, 319)	0.954	411 (347, 462)	395 (348, 460)	0.624
B. Propensity matched data comparisons										
*N*		71	71		64	64		23	23	
Age	Median (25/75 interval)	63 (58, 69)	65 (59, 70)	0.482	64 (58, 69)	66 (60, 70)	0.309	72 (67, 78)	72 (67, 78)	1
Height	Median (25/75 interval)	174 (171, 179)	177 (171, 182)	0.263	175 (170, 180)	176 (172, 180)	0.845	172 (167, 178)	173 (167, 177)	0.675
Weight	Median (25/75 interval)	88 (79, 100)	88 (83, 101)	0.481	88 (80, 99)	89 (81, 102)	0.945	80 (65, 93)	76 (63, 85)	0.893
ASA	Median (25/75 interval)	2 (2, 3)	2 (2, 3)	1	2 (2, 3)	2 (2, 3)	0.746	3 (3, 3)	3 (3, 3)	1
IV Fluid (ml)	Median (25/75 interval)	1900 (1522, 2300)	1700 (1400, 2000)	0.185	1900 (1572, 2425)	1625 (1350, 2000)	0.186	3820 (2766, 4080)	2888 (2244, 3419)	0.0286
EBL (ml)	Median (25/75 interval)	152 (100, 200)	100 (100, 185)	0.962	100 (100, 200)	100 (100, 150)	0.689	225 (150, 625)	375 (107, 500)	0.782
Operative time (minutes)	Median (25/75 interval)	332 (294, 360)	300 (258, 327)	*< 0.001*	299 (241, 338)	286 (228, 326)	0.911	417 (377, 462)	405 (376, 458)	0.485

A: Unmatched data comparisons: age (years), height (cm), weight (kg), ASA = American Society of Anesthesiologists physical status classification system, IV = total intravenous fluids (milliliters), EBL = estimated blood loss (milliliters), operative time (minutes). B: Propensity matched data comparisons: age (years), height (cm), weight (kg), IV = total intravenous fluids (milliliters), EBL = estimated blood loss (milliliters), operative time (minutes)

**Table 4:** Outcome data for impact evaluation of perioperative hospitalist service for urologic procedures

	Prostatectomy pre-program	Prostatectomy post-program	Nephrectomy pre-program	Nephrectomy post-program	Cystectomy pre-program	Cystectomy post-program
A. Unmatched data comparisons						
Primary outcome						
*N*	72	125	65	90	26	46
Length of stay (days) median (25/75 interval)	1.5 (1–2)	1 (1–2)	4 (3–5)	3 (2–3)	9 (8–13.5)	7 (5.25–8.75)
Bed days saved (mean difference - days)		0.27		1.3		3.1
Length of stay *p* value		0.058		< 0.001		< 0.001
Secondary outcome						
Percentage of total complications (total complications/amount of opportunities for complications)	4.8%	1.5%	9.8%	6.3%	7.0%	3.6%
AKI	2.8%	0.8%	4.6%	3.3%	7.7%	2.2%
DVT	0	0	0	0	0	0
Myocardial infarction	4.2%	1.6%	4.6%	4.4%	0	0
Pneumonia	0	0.8%	1.5%	1.1%	3.8%	0
Pulmonary embolus	0	0	1.5%	0	0	0
Sepsis	0	0	0	0	0	0
Stroke	0	0	0	0	0	2.2%
Surgical site infection	0	0	0	0	0	0
UTI	0	0	0	0	0	2.2%
Erectile dysfunction	0	0	0	0	0	0
Ileus	45.8%	13.6%	95.3%	57.8%	65.4%	32.6%
Ileus Incidence *p* value		< 0.001		< 0.001		< 0.001
Return of flatus (POD) median (25/75 interval)	2 (1–2)	2 (1–2)	4 (3–4)	3 (3–4)	5 (5–6)	4 (3–5)
*p* value		0.422		0.029		< 0.001
Percentage of subjects with consultations	5.6%	6.4%	29.3%	12.2%	38.5%	32.6%
% Change direct cost (standard error of estimate)		− 0.12% (2.75)		−14.55% (3.00)		−11.83% (7.52)
*p* value		0.555		0.306		0.172
B. Propensity matched data comparisons						
Primary outcome						
*N*	71	71	64	64	23	23
Length of stay (days) median (25/75 interval)	2 (1–2)	1 (1–2)	4 (3–5)	3 (2–3)	9 (8–13.5)	7 (5–10)
Bed says saved (mean difference - days)		0.44		1.3		3.6
Length of stay *p* value		0.009		< 0.001		0.009
Secondary outcome						
Percentage of total complications (total complications/amount of opportunities for complications)	4.6%	1.0%	9.8%	6.4%	7.1%	2.0%
AKI	2.8%	1.4%	4.7%	3.1%	8.7%	4.3%
DVT	0	0	0	0	0	0
Myocardial infarction	2.8%	1.4%	4.7%	6.3%	0	0
Pneumonia	0	1.4%	1.6%	1.6%	4.3%	0
Pulmonary embolus	0	0	1.6%	0	0	0
Sepsis	0	0	0	0	0	0
Stroke	0	0	0	0	0	4.3%
Surgical site infection	0	0	0	0	0	0
UTI	0	0	0	0	0	4.3%
Erectile Dysfunction	0	0	0	0	0	0
Ileus	45%	7%	95.3%	59.4%	65.2%	8.7%
Ileus Incidence *p* value		< 0.001		< 0.001		< 0.001
Return of flatus (POD) median (25/75 interval)	2 (1–2)	2 (1–2)	4 (3–4)	3 (3–4)	5 (5–6.75)	4 (3–4.5)
*p* value		0.854		0.005		< 0.001
Percentage of subjects with consultations	5.6%	2.8%	29.7%	17.2%	34.8%	13%
% Change direct cost (standard error of estimate)		− 5.59% (2.54)		− 15.88% (3.18)		− 22.15% (6.21)
*p* value		0.317		0.277		0.075

A: Unmatched data comparisons. B: Propensity matched data comparisons: *POD* post-operative day, *AKI* acute kidney injury, *DVT* deep venous thrombosis, *UTI* urinary tract infection

### Primary outcome

Length of stay was significantly reduced (*p* <  0.01) from the pre-PHS service to the post-PHS service implementation for all three surgical procedures with propensity matching (Table 4B). Unmatched analysis demonstrated statistically significant reduction in LOS (*p* <  0.001) for cystectomy and nephrectomy procedures (Table 4A). Cystectomies demonstrated the largest percent reduction in LOS.

### Secondary outcome

Comparisons of complications between pre- and post-PHS implementation are shown in Table 4A for unmatched data and in Table 4B for propensity matched data. Significant reductions in overall complication rates and incidence of ileus were observed for all surgical procedures post-PHS implementation (*p* <  0.0001), for both datasets. Similarly, return of bowel function (flatus) was also significantly reduced (*p* <  0.03) post-PHS implementation for both cystectomy and nephrectomy procedures under both methods of data analysis. The majority of the reductions in complications were secondary to an improvement in the incidence of ileus for all surgical procedures. Additionally, decreases in total direct costs and frequency of consultations were also observed for the cystectomy and nephrectomy groups (Table 4A and B). Finally, a significant difference in the average bed days between pre and post-PHS implementation was also observed for all surgical procedures.

## Discussion

Under the conditions of this quality improvement project, we found that implementation of *perioperative hospitalist service* (1) reduced hospital length of stay, (2) reduced hospital costs, (3) reduced postoperative complications, and (4) resulted in fewer consultations. These findings align with recent studies demonstrating the utility of coordinated care efforts to improve the perioperative experience. Importantly, this study demonstrated the utility of anesthesiologists to not only facilitate these coordinated care efforts but also provide postoperative medical management. This role has traditionally been designated to a hospitalist, which has not often been viewed as a clinical role for anesthesiologists in the United States.

### Anesthesiologists as perioperative hospitalists

With the rapidly changing landscape of perioperative care, emphasis has been placed on anesthesiologists to diversify their practice paradigms to maintain the specialty’s significance in medicine (Anesthesia MRRftTFoFPo 2007). In 2008, the American Board of Anesthesiologists implemented perioperative medicine as a milestone for residency training. Furthermore, a residency curriculum roadmap for perioperative medicine has recently been developed, incorporating competencies fundamental to perioperative medicine (Alem et al. 2016). However, despite the call to widen the scope of training and practice to include more perioperative care, the majority of anesthesiologists in both academic and private practice still choose to limit involvement in perioperative care to the immediate postoperative phase (i.e., post-anesthesia care-PACU), allowing other medical specialties to serve as perioperative hospitalists. In so doing, a significant opportunity that currently exists to assume a leadership role in perioperative care coordination efforts and clinical system redesign is squandered. This study demonstrates that anesthesiologists can be trained to serve as perioperative hospitalists, providing high-quality comprehensive postoperative care. This is of particular importance within academic institutions, where the variability in physician house staff training level and service assignments change frequently, destabilizing clinical management and planning.

This study suggests that perioperative care coordination and postoperative medical management are within the skill set of an anesthesiologist. The utility of intensivists providing daily rounds on post-surgical patients has been demonstrated to provide both clinical and economic benefits (Hanson 3rd et al. 1999; Macario et al. 1995). As many intensivists are anesthesiologists, it seems intuitive to believe that an anesthesiologist can learn the additional material and skills needed to provide post-surgical non-ICU hospitalist care. Indeed, three out of the five PHS team members presented in this study were anesthesiology intensivists. Importantly, however, the other two team members were general anesthesiologists. These two PHS members received additional training in the areas previously identified that are not considered to be part of core anesthesiology training, as well as with any additional areas identified during the initial team planning meetings. Training was performed as small group didactic sessions, which included the surgeons, nurse practitioners, and PHS physician members who had attested to the willingness to acquire the additional training necessary to provide postoperative hospitalist care.

While the role of anesthesiologists serving as hospitalists is debatable for a multitude of reasons (level of interest, financial implications, medical training), this study demonstrates that the concept is feasible. The use of the anesthesiologist functioning as a perioperative hospitalist demonstrates that the training and expertise of anesthesiologists can be further utilized to provide and direct postoperative care activities within a multidisciplinary team model framework. Importantly, however, this study does not evaluate the ability of the clinical benefit demonstrated in this project to balance the financial cost of running the PHS service described in this study.

Nonetheless, this study highlights the potential for expansion of the scope of practice of anesthesiologists. The results of this study are not intended to suggest that the concept of an anesthesiologist functioning in the role of a perioperative hospitalist must be embraced by all anesthesiologists; rather, this study supports the viability of the novel approach to improving perioperative care for those individuals interested in the promising field of perioperative hospital medicine.

### Limitations

This study has several limitations. As with all quality improvement studies in comparison to randomized trials, there is a reduced ability to associate causal connection between the intervention and outcome. Propensity analysis was conducted to adjust for baseline difference across years. After propensity analysis, our results indicated the sustained improvement in outcome was effective in demonstrating clinical value. Importantly, all data was analyzed retrospectively with the same data collection methodology. During this review, no change in surgical practice intraoperatively was noted, yet a decrease in surgical time was noticed for prostatectomy procedures. One explanation could be improved workflow intraoperatively between nursing, surgery, surgical scrubs, and anesthesiology; however, this result demonstrates the limitations in our retrospective review. Similarly, complications were dependent on documentation by the health care providers in the patient care clinical documents. Also, secondary to the significant allocation of resources needed to support this project, the PHS attending only provided in person consultation on weekends and holidays per request by the urology service. Moreover, the sample of physicians involved in the PHS service was small relative to the size of the department (5 out of 63 faculty). Importantly, the number of faculty members that were involved in the program was determined by the amount of resources needed to provide service coverage (as described above) and by the department members who expressed interest in the hospitalist concept; all PHS member involvement in the program was voluntary. The study sample size was determined by the maximal amount of time approved by the anesthesiology department (2 years) to evaluate the QI initiative. Thus, no prospective power analysis was performed. Finally, the surgical study population reported in this study was limited to three urologic procedures and seven surgeons. All care pathways and postoperative goals were identified with this patient group in mind.

## Conclusion

This study supports the literature demonstrating the positive role of hospitalists to improve patient care through facilitation of medical management and coordination of care efforts. Importantly, this investigation demonstrates the ability of anesthesiologists to safely function in the role of perioperative hospitalist, both in regard to facilitating coordinate care efforts as well as with medical management.

## Additional file

Additional file 1: Propensity balance tables PHS study. (DOCX 166 kb)

## Abbreviations

ERAS: Enhanced Recovery After Surgery
GRACE: Good Research for Comparative Effectiveness
IRB: Institutional Review Board at Loma Linda University
LOS: Length of stay
PHS: Perioperative hospitalist service
PSH: Perioperative surgical home
QI: Quality improvement
SHM: Society of Hospitalist Medicine
SQUIRE: Standards for Quality Improvement Reporting Excellence
U.S.: United States
