# Supplementary material for: Anesthesiologists as perioperative hospitalists and outcomes in patients undergoing major urologic surgery: a historical prospective, comparative effectiveness study
Source: Perioper Med (Lond). 2018 Jun 19;7:13. doi: 10.1186/s13741-018-0090-y (PMC6009851; doi:10.1186/s13741-018-0090-y)
Supplement: Supplementary file 1 — Propensity balance tables PHS study. (DOCX 166 kb) [file 13741_2018_90_MOESM1_ESM.docx]

**Prostatectomy**:

Summary of balance for all data:

| Means Treated Means Control SD Control Std. Mean Diff. eQQ Med eQQ Mean eQQ Max |
| --- |
| distance 0.6388 0.6270 0.0508 0.2172 0.056 0.0442 0.1084 |
| Age 65.2560 63.4722 7.3081 0.2220 0.056 0.0442 0.1084 |

Summary of balance for matched data:

| Means Treated Means Control SD Control Std. Mean Diff. eQQ Med eQQ Mean eQQ Max |
| --- |
| distance 0.6350 0.6291 0.0480 0.1083 0.0282 0.0296 0.0845 |
| Age 64.6197 63.7606 6.9353 0.1069 0.0282 0.0296 0.0845 |

Percent Balance Improvement:

| Std. Mean Diff. eQQ Med eQQ Mean eQQ Max |
| --- |
| distance 50.1311 49.6982 33.0323 22.0734 |
| Age 51.8351 49.6982 33.0323 22.0734 |


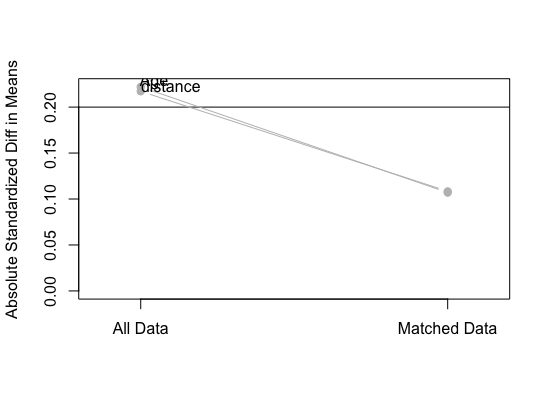


| Sample sizes: Control Treated |
| --- |
| All 72 125 |
| Matched 71 71 |
| Unmatched 1 54 |
| Discarded 0 0 |

**Nephrectomy:**

Summary of balance for all data:

| Means Treated Means Control SD Control Std. Mean Diff. eQQ Med eQQ Mean eQQ Max |
| --- |
| distance 0.5831 0.5773 0.0159 0.1157 0.0333 0.0415 0.1333 |
| Weight 96.6600 86.1292 19.1668 0.1065 0.0265 0.0334 0.1179 |
| Height 173.5400 167.6738 10.6313 0.0647 0.0231 0.0270 0.0846 |

Summary of balance for matched data:

| Means Treated Means Control SD Control Std. Mean Diff. eQQ Med eQQ Mean eQQ Max |
| --- |
| distance 0.5800 0.5768 0.0157 0.0634 0.0625 0.0609 0.1250 |
| Weight 89.6891 85.6094 18.8508 0.0413 0.0625 0.0642 0.1562 |
| Height 168.2484 167.5437 10.6631 0.0078 0.0625 0.0636 0.1719 |

Percent Balance Improvement:

| Std. Mean Diff. eQQ Med eQQ Mean eQQ Max |
| --- |
| distance 45.2326 -87.5000 -46.8952 6.2500 |
| Weight 61.2594 -135.8871 -92.2849 -32.4728 |
| Height 87.9872 -170.8333 -135.4335 -103.1250 |


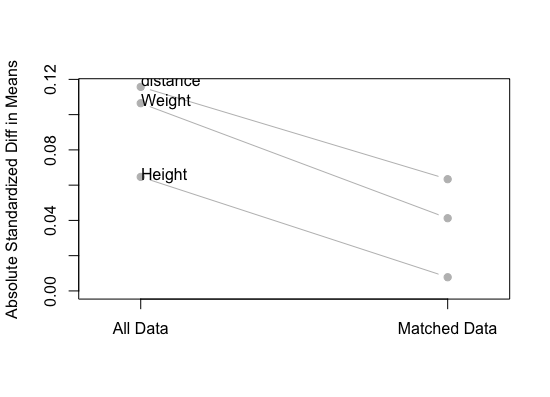


| Sample sizes: Control Treated |
| --- |
| All 65 90 |
| Matched 64 64 |
| Unmatched 1 26 |
| Discarded 0 0 |

**Cystectomy:**

Summary of balance for all data:

| Means Treated Means Control SD Control Std. Mean Diff. eQQ Med eQQ Mean eQQMax |
| --- |
| distance 0.6479 0.6230 0.0704 0.3125 0.0895 0.0941 0.2224 |
| Age 71.2391 68.8077 14.0940 0.2214 0.0351 0.0392 0.1104 |
| Height 167.4428 172.8885 9.2222 -0.2052 0.0661 0.0702 0.1990 |

Summary of balance for matched data:

| Means Treated Means Control SD Control Std. Mean Diff. eQQ Med eQQ Mean eQQ Max |
| --- |
| distance 0.6391 0.6335 0.0438 0.0701 0.0435 0.0589 0.2174 |
| Age 69.9565 71.5652 9.1494 -0.1465 0.0435 0.0518 0.1304 |
| Height 170.6565 172.8609 8.7485 -0.0831 0.0870 0.0747 0.2174 |

Percent Balance Improvement:

| Std. Mean Diff. eQQ Med eQQ Mean eQQ Max |
| --- |
| distance 77.5797 51.4019 37.3989 2.2556 |
| Age 33.8377 -23.8095 -32.3415 -18.1818 |
| Height 59.5208 -31.6456 -6.3988 -9.2437 |


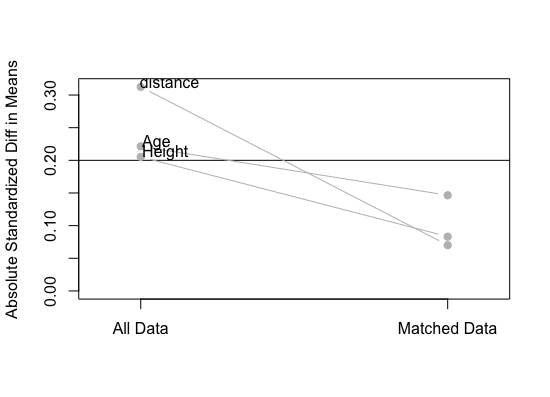


| Sample sizes: Control Treated |
| --- |
| All 26 46 |
| Matched 23 23 |
| Unmatched 3 23 |
| Discarded 0 0 |
